# Update in pediatric myopia treatment strategies

**Published:** 2020

**Authors:** Silvia Țone, Irina Andreea Niagu, Ștefan Tudor Bogdănici, Camelia Margareta Bogdănici

**Affiliations:** * Discipline of Ophthalmology, “Grigore T. Popa” University of Medicine and Pharmacy, Iași, Romania; ** “Stereopsis” Ophthalmological Clinic, Iași, Romania

**Keywords:** children, myopia, treatment

## Abstract

Pediatric myopia affects more and more children in Asia, USA and other countries. There is no standard protocol for the therapy but pediatric ophthalmologists try to decrease myopia progression using different methods. Myopia in children is more and more frequent and the onset age has decreased over time, leading to a greater chance to progression into high diopters for spectacles or contact lenses and also other ocular complications. Regarding this issue, the aim of this paper was to underline the new therapeutic regimens for correcting and slowing pediatric myopia progression.

## Introduction

Pediatric myopia affects approximately 23% of the worldwide population with a prevalence between 0.9% to 3.1% [**1**]. In the past years, the prevalence of this condition has reached dramatic proportions in East Asia [**2**,**3**]. The ocular morbidity is linked to axial growth, representing an important cause of permanent vision disfunction [**4**,**5**]. Myopia usually manifests in childhood, typically between the ages of 7 to 10 years old, and after that, progresses over the next 10 to 15 years [**6**]. The prevalence of severe myopia is increasing in urban areas of Asia [**7**] and recent studies showed a similar situation in the USA [**8**] and many other populations [**9**].

Because the prevalence increases and the age of onset decreases, the condition has more time to progress and the risk of high myopic errors and other ocular complications to occur is more probable [**10**]. Because of that, treatment to limit the progression would be beneficial [**11**].

However, in the absence of an official treatment protocol, there is a significant variation in treatment patterns among pediatric ophthalmologists in order to decrease myopia progression. The aim of this paper was to evaluate the new therapeutic strategies for correcting and limiting myopia evolution in this age group.

## Treatment to Slow the Progression of Myopia

**Single Vision Lenses and Refractive Under correction**

Single Vision Lenses (SVLs) represent the most often used therapeutic option for treating myopia. Although this method helps improving the visual acuity, it does not decrease the myopic evolution or ocular axial growth. Moreover, it has been showed that the use of SVLs might favor myopia progression and axial elongation [**12**,**13**]. 

From that point it was taught that undercorrection of myopic eyes with eye glasses should slow myopic progression. This concept of under correction was also shown in a couple of infant animal studies. Unfortunately, this finding did not work on children. Recent studies comparing the effects of under and full correction of myopia, explained that undercorrection might actually increase myopia progression [**14**]. Chung et al. study, which compared the effects of under and full correction spectacles, was interrupted after two years due to significant progression of the disease in the undercorrected group (0.23D) and a 30% rate of axial growth [**15**].

It is not clear why undercorrected eyes progress more quickly than normal but undercorrection is definitely not an efficient treatment strategy. Moreover, it is recommended that patients with progressive myopia should be regularly evaluated. 

Another treatment strategy is represented by part-time spectacle wear. There is a three-year study that analyzed the effects of different patterns of lens wear on myopia progression showing no benefits after part-time lens wear in myopia evolution [**16**].

## Bifocal Lenses and Progressive Additional Lenses (PALs)

Another studied therapeutic alternative against the progression of myopia is the use of bifocals lenses or PALs. By reducing the accommodation associated with near viewing tasks, they eliminate the delay in accommodation and the consecutive hyperopic defocus at the fovea [**17**,**18**]. The largest study with this type of lens was the COMET study (*Correction of Myopia Evaluation Trial*), which aimed to evaluate whether PALs limit the evolution of myopia versus conventional SVLs. The study explained that patients with more important delays of accommodation and near esophoria could benefit from this treatment, compared to the others. Because of the limited period of time, the study did not find clinically significant results [**19**].

A similar study was conducted in Japan and included patients with ages between 6 and 12 years old, with myopia from −1.25 to −6.0 D. The participants wore one type of spectacle lens for the first half period of the study, and then another type of eyeglasses for the second half. Results showed improvement of 0.17 D at 18 months for patients who used PALs first. Early treatment with PALs could have been more efficient than SVLs in these patients. Similar to the *COMET trial*, patients with higher accommodative lags and those with esophoria or near orthophoria had better results after therapy than the ones with smaller lags (0.61 D vs. 0.15 D) or those with exophoria (0.55 D vs. 0.18 D) [**20**]. 

## MyoVision Lenses

These lenses represent a single vision lens with specific myopia management design that is effective in preventing myopic evolution. The periphery of the lens is responsible for myopia control, whereas the central part provides a sharp vision correcting myopia. With SVLs, the central zone works fine but the margin of the lens often projects the image behind the visual area. This can lead to a signal to the eye to elongate, favoring the myopic evolution. MyoVision Lenses active zone in the margin corrects this defocus and in consequence can help limit the progression.

A 12-month wearer efficacy trial amongst 210 Chinese school children resulted in limiting the myopic evolution by an average of 30% in children between 6 and 12 years old, with at least one parents with myopia. A statistically significant reduced progression of 0.29 D was identified in eyes wearing a new rotationally asymmetric lens versus control lenses [**21**].

More recently, another 2 years study was conducted in Japan on 207 patients with ages between 6 and 12 years old with spherical equivalent refractions ranging from -1.5 to -4.5 diopters and with at least one parent diagnosed with myopia. This study could not verify the therapeutic effect of these kind of lenses in limiting myopic evolution in children from Japan. Additional studies are needed to design lenses that can lower peripheral hyperopic defocus individually and to analyze the benefit of these lenses in preventing myopic evolution [**22**].

## Peripheral Retinal Defocus

According to literature, peripheral retina and peripheral vision are both associated with the development of myopia [**23**]. The lack of central vision is not associated with myopia, indicating that peripheral vision has a more significant role. 

In the past, a soft contact lens called ‘*Defocus Incorporated Soft Contact*’ (DISC) lens was created for myopic control. Based on the same myopic defocus mechanism, the *Defocus Incorporated Multiple Segments* (DIMS) spectacle lens was designed, providing the same optical stimulus as the DISC lens. Patients using DIMS spectacle lenses had 52% lower myopic progression and a 62% lower axial growth over 2 years versus SV lenses wearers. This treatment is easy to apply and is the least invasive when compared to pharmacological therapies or contact lenses [**24**].

## MiSight Contact Lenses (cooperVision)

These lenses represent a disposable, soft lens designed for one-time use like classic contact lenses, and are FDA approved. The efficiency and protection of the lens were examined during a 3-year randomized controlled clinical study conducted on 135 children with ages between 8 and 12 years old. This type of lenses induces peripheral myopic defocus. The contact lenses have a central part of full myopic power and also a peripheral plus “rings” to defocus. According to this study, myopic evolution is greatly reduced by the MiSight soft contact lens [**25**].

Ruiz-Pomeda et al. conducted a 2-year clinical trial analyzing myopia progression with the MiSight lens versus single-vision spectacles. The mean change in axial elongation at the 12-month follow-up was 0.12 mm for the MiSight lens and 0.24 mm for the other group, and at the 24-month follow-up, the variation in axial growth was −0.16 mm [**26**]. Considering all of that, MiSight contact lens may be an alternative for slowing the progression of myopic errors.

## Corneal Reshaping Therapy

**Rigid Gas Permeable Contact Lens and Orthokeratology (Orthok)**

Contact lenses represent an alternative for treating myopia in children, slowing down its evolution and limiting ocular axial growth. Rigid gas permeable contact lens (RGPCLs), Orthok contact lens and soft bifocal contact lens are available for this type of treatment. The *Contact Lens and Myopia Progression* (CLAMP) study enrolled one hundred and sixteen children with ages between 8 and 12 years old, who were asked to wear either RGP or soft contact lenses for three years. The participants had to complete a run-in period successfully prior to the enrolment, to exclude those who could not adapt to RGP. Results showed a statistically significant difference in the 3-year progression of myopia in the RGP vs. soft lens group and curvature of the cornea steepened less over three years in the RGP group (0.62 ± 0.60 D) versus the soft lens group (0.88 ± 0.57 D). Instead, axial length was not meaningfully different between the groups and explained that slowed myopia progression was determined by corneal flattening. Overall, the trial showed that RGP may be an alternative, but should not be recommended mainly for myopic control [**27**].

Another study analyzed 100 myopic patients with ages between 8 and 13 years old, who were ﬁtted with RGP contact lenses and 20 patients who used spectacles. The evolution of myopia was signiﬁcantly different with 0.48D progression for RGPCLs group in comparison with the −1.53 D progression for spectacles wearers group. The temporary limitation of evolution was also due to the altering of the corneal curvature and not the ocular axial elongation [**28**].

*The Longitudinal Orthokeratology Research in Children* (LORIC) study was designed to determine if wearing RGP lenses during the night helped with the axial length evolution and the myopic progression. Thirty-five children wore special designed lenses and were compared to another group wearing SVLs, for two years. There were major differences between the groups that showed a 0.25 mm higher axial length in the control group [**29**]. Berkeley Orthokeratology study, conducted on 80 subjects who used orthokeratology versus a control group, identified a signiﬁcantly higher myopic reduction in orthokeratology group versus the other group, but unfortunately the study had minimal clinical signiﬁcance [**30**]. 

These are promising studies that demonstrate the demand for a well-designed study on what is now called corneal reshaping therapy with sufficient subject numbers, adequate control groups and long-term follow-ups to obtain clinical importance. Of course, the complication of the lenses, like microbial keratitis, compliance of the patients and family and participation to follow-ups must be taken into consideration to fully evaluate this treatment for slowing myopia progression.

**Fig. 1:**
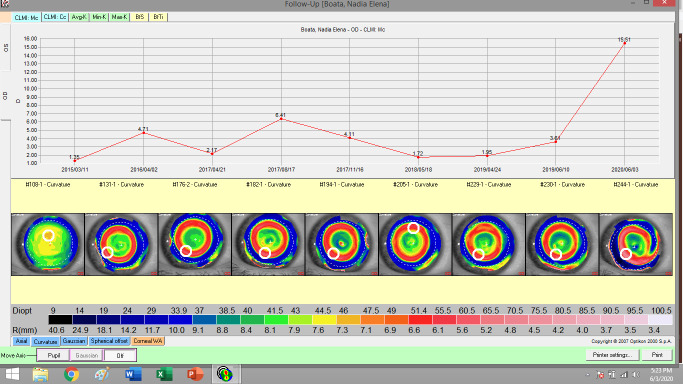
Topography of right eye in evolution (personal case)

**Fig. 2:**
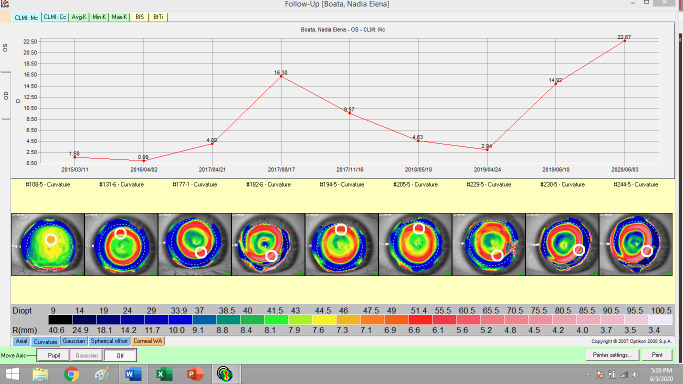
Topography of left eye in evolution (personal case)

The night contact lenses used in orthokeratology are manufactured after the interpretation of the corneal topography (**Fig. 1**,**2**). The red ring indicates that the night time lens had the expected effectiveness. Orthokeratology improved the quality of visual acuity and life. The risk of amblyopia can be lowered by using orthokeratology in children with myopic anisometropia [**31**].

Better results can be achieved in myopic evolution by using two different therapies combined instead of each one on its own. Nozomi Kinoshita and colleagues conducted a study that compared the use of both orthokeratology and atropine versus the use of only orthokeratology. The results showed that the combination of the therapies had a synergistic effect being 28% to 38 % more efficient in myopic progression [**32**].

## Pharmaceutical Agents

Topical *atropine* has demonstrated statistically and clinically significant reductions in myopic progression, being supported by a lot of studies. Shih et al. conducted a randomized study on children with ages between 6 and 13 years old, comparing the use of 0.5% atropine and multifocal glasses, multifocal glasses alone or SVLs alone. The study reported better results in the atropine group (0.41D vs. 1.19 D and 1.40 D) [**33**].

Chua et al. reported similar results in a two-year study of 400 participants, 6 to 12-year-old myopic children in Singapore [**34**]. The study compared an atropine group with a placebo-control group. The 1-year washout period showed important rebound phenomenon in the refraction in the axial elongation and it was efficient at the end of the two-year therapy phase. This study was followed by a 5-year clinical trial that analyzed the efﬁcacy of smaller doses of atropine on limiting the myopic evolution, while also minimizing the adverse effects. Patients received either 0.5% or 0.1% or 0.01% atropine for 2 years, followed by a 1-year washout period. The conclusion was that all 3 doses of atropine remained efficient in lowering myopic progression and the concentration of 0,01% had the least side effects.

Although atropine is used in many countries in Asia for limiting myopic progression, it is rarely used in the USA and Europe because of both short-term and long-term side effects. The side effects associated with atropine are photophobia, cycloplegia, allergic dermatitis, increased intraocular pressure, cataract and angle closure glaucoma and should not be recommended for long-term use.

*Pirenzepine* is used as an alternative treatment for pediatric myopia, less possible to cause mydriasis or cycloplegia and has fewer side effects. Pirenzepine may have a positive role in slowing down myopic progression in children [**35**].

## Conclusions

Although we have clarified many uncertainties regarding the reduction of myopic progression in children, many questions are still unanswered. Children and their parents should always discuss the risks and benefits of the treatment with their ophthalmologist. Far more studies need to be conducted to solve all the questions, so that we can improve eye care for children and eventually prevent or maintain a lower amount of myopia, which may decrease the risk of other ocular complications.

**Disclosures**

None.

**Conflicts of interest**

None.
